# Supplementary material for: A systematic review and evidence synthesis of non-medical triage, self-referral and direct access services for patients with musculoskeletal pain
Source: PLoS One. 2020 Jul 6;15(7):e0235364. doi: 10.1371/journal.pone.0235364 (PMC7337346; doi:10.1371/journal.pone.0235364)
Supplement: S1 Table — (DOCX) [file pone.0235364.s002.docx]

S1 Table: Detailed Search Strategy (Medline)

| \| 1 \| triage/ \| \| --- \| --- \| \| 2 \| triag$.ti,ab,kw. \| \| 3 \| (direct$ adj3 access$).ti,ab,kw. \| \| 4 \| self refer$.ti,ab,kw. \| \| 5 \| refer$ themsel$.ti,ab,kw. \| \| 6 \| (patient$ adj3 initiate$ adj3 refer$).ti,ab,kw. \| \| 7 \| (first adj3 (contact or call)).ti,ab,kw. \| \| 8 \| (NHSdirect or NHS direct).ti,ab,kw. \| \| 9 \| Telemedicine/ \| \| 10 \| (telemedicine or tele medicine).ti,ab,kw. \| \| 11 \| (telehealth$ or tele health$).ti,ab,kw. \| \| 12 \| teletriag$.ti,ab,kw. \| \| 13 \| (walk in adj3 (clinic* or service* or centre* or center*)).ti,ab,kw. \| \| 14 \| (interface adj3 (clinic* or service* or centre* or center*)).ti,ab,kw. \| \| 15 \| or/1-14 \| \| 16 \| exp pain/ \| \| 17 \| (pain$ or ache$1 or aching).ti,ab,kw. \| \| 18 \| (stiff or stiffness or impingement$).ti,ab,kw. \| \| 19 \| (strain$ or sprain$).ti,ab,kw. \| \| 20 \| or/16-19 \| \| 21 \| exp Musculoskeletal System/ \| \| 22 \| (muscle$ or tendon$ or ligament$).ti,ab,kw. \| \| 23 \| exp Lower extremity/ \| \| 24 \| (knee or knees).ti,ab,kw. \| \| 25 \| hip$1.ti,ab,kw. \| \| 26 \| ankle$1.ti,ab,kw. \| \| 27 \| (foot or feet).ti,ab,kw. \| \| 28 \| exp Upper extremity/ \| \| 29 \| shoulder$.ti,ab,kw. \| \| 30 \| elbow$1.ti,ab,kw. \| \| 31 \| wrist$1.ti,ab,kw. \| \| 32 \| hand$1.ti,ab,kw. \| \| 33 \| (arm$1 or forearm$1).ti,ab,kw. \| \| 34 \| exp back/ or back.ti,ab,kw. \| \| 35 \| neck/ or neck.ti,ab,kw. \| \| 36 \| (spine or spinal).ti,ab,kw. \| \| 37 \| lumbar.ti,ab,kw. \| \| 38 \| cervical.ti,ab,kw. \| \| 39 \| joint$.ti,ab,kw. \| \| 40 \| rotator cuff.ti,ab,kw. \| \| 41 \| or/21-40 \| \| 42 \| 20 and 41 \| \| 43 \| exp Musculoskeletal Diseases/ \| \| 44 \| (musculoskeletal or MSK).ti,ab,kw. \| \| 45 \| arthriti$.ti,ab,kw. \| \| 46 \| bursitis.ti,ab,kw. \| \| 47 \| capsulitis.ti,ab,kw. \| \| 48 \| (tendonitis or tendinitis).ti,ab,kw. \| \| 49 \| tendinopath$.ti,ab,kw. \| \| 50 \| tenosynovitis.ti,ab,kw. \| \| 51 \| (chronic adj3 pain).ti,ab,kw. \| \| 52 \| ((multisite or "multi site") adj3 pain).ti,ab,kw. \| \| 53 \| pain syndrome$.ti,ab,kw. \| \| 54 \| osteoarthr$.ti,ab,kw. \| \| 55 \| arthralgi$.ti,ab,kw. \| \| 56 \| arthrosis.ti,ab,kw. \| \| 57 \| rheumatology/ \| \| 58 \| rheumat$.ti,ab,kw. \| \| 59 \| (joint$ adj3 disease$).ti,ab,kw. \| \| 60 \| myositis.ti,ab,kw. \| \| 61 \| myopath$.ti,ab,kw. \| \| 62 \| myalgia$.ti,ab,kw. \| \| 63 \| whiplash$.ti,ab,kw. \| \| 64 \| exp "Sprains and Strains"/ \| \| 65 \| impingement syndrome$.ti,ab,kw. \| \| 66 \| anterior cruciate ligament injuries/ or tibial meniscus injuries/ \| \| 67 \| Whiplash Injuries/ \| \| 68 \| rotator cuff injuries/ \| \| 69 \| or/42-68 \| \| 70 \| physical therapist/ \| \| 71 \| physical therap$.ti,ab,kw. \| \| 72 \| (physio$1 or physiotherap$).ti,ab,kw. \| \| 73 \| Osteopathic Physicians/ \| \| 74 \| osteopath$.ti,ab,kw. \| \| 75 \| Chiropractic/ \| \| 76 \| chiropract$.ti,ab,kw. \| \| 77 \| ((extend$ or expand$ or advance$) adj3 (scope$ or practice$ or role$)).ti,ab,kw. \| \| 78 \| exp Nurse Practitioners/ \| \| 79 \| (nurse adj2 (practitioner$ or practice)).ti,ab,kw. \| \| 80 \| Advanced Practice Nursing/ \| \| 81 \| (physician$ adj (associate$ or assistant$)).ti,ab,kw. \| \| 82 \| or/70-81 \| \| 83 \| primary assess$.ti,ab,kw. \| \| 84 \| ((profession$ or role$ or task$ or doctor$ or GP) adj3 substitut$).ti,ab,kw. \| \| 85 \| 83 or 84 \| \| 86 \| 15 and 69 \| \| 87 \| 15 and 82 \| \| 88 \| 82 and 85 \| \| 89 \| (physiodirect or physio direct).ti,ab,kw. \| \| 90 \| or/86-89 \| \| 91 \| exp animals/ not humans/ \| \| 92 \| 90 not 91 \| |
| --- | --- | --- | --- | --- | --- | --- | --- | --- | --- | --- | --- | --- | --- | --- | --- | --- | --- | --- | --- | --- | --- | --- | --- | --- | --- | --- | --- | --- | --- | --- | --- | --- | --- | --- | --- | --- | --- | --- | --- | --- | --- | --- | --- | --- | --- | --- | --- | --- | --- | --- | --- | --- | --- | --- | --- | --- | --- | --- | --- | --- | --- | --- | --- | --- | --- | --- | --- | --- | --- | --- | --- | --- | --- | --- | --- | --- | --- | --- | --- | --- | --- | --- | --- | --- | --- | --- | --- | --- | --- | --- | --- | --- | --- | --- | --- | --- | --- | --- | --- | --- | --- | --- | --- | --- | --- | --- | --- | --- | --- | --- | --- | --- | --- | --- | --- | --- | --- | --- | --- | --- | --- | --- | --- | --- | --- | --- | --- | --- | --- | --- | --- | --- | --- | --- | --- | --- | --- | --- | --- | --- | --- | --- | --- | --- | --- | --- | --- | --- | --- | --- | --- | --- | --- | --- | --- | --- | --- | --- | --- | --- | --- | --- | --- | --- | --- | --- | --- | --- | --- | --- | --- | --- | --- | --- | --- | --- | --- | --- | --- | --- | --- | --- | --- | --- |
