# Supplementary material for: A systematic review and evidence synthesis of non-medical triage, self-referral and direct access services for patients with musculoskeletal pain
Source: PLoS One. 2020 Jul 6;15(7):e0235364. doi: 10.1371/journal.pone.0235364 (PMC7337346; doi:10.1371/journal.pone.0235364)
Supplement: S2 Table — (DOCX) [file pone.0235364.s003.docx]

S2 Table: Detailed Eligibility Criteria

|  | **Inclusion criteria** | **Exclusion criteria** |
| --- | --- | --- |
| **Study design** | Mixed designs  Including: RCTs, before-after designs, comparative cohort, cross-sectional surveys, and qualitative studies | Case studies only  Editorials/ protocols/ scientific meeting abstracts for which full data could not be obtained. |
| **Participants and conditions of interest** | Adults ≥18yrs with MSK condition  NB: Explicit focus on MSK was required at full text selection. At least 50% of the patient population was described as MSK **or** sub-group outcome data were reported separately for MSK populations in the paper. | Children <18 years  Non primarily MSK conditions, or where co-morbidity exists **AND** MSK is not the main reason for health care consultation. |
| **Interventions** | Musculoskeletal triage or patient direct access/self-referral services led by non-medical health professional (allied health professional).  NB: telemedicine studies were only included if they concerned triage or an algorithm system for triage. | NB: Studies using telemedicine for self-management, to supplement or to replace face-to-face follow-up consultations were excluded. Telemedicine studies involving physicians were also excluded. |
| **Comparisons or control groups** | Usual care/GP led services/no comparisons | n/a |
| **Outcomes of interest** | Eligible study must include at least 1 outcome in any of the following 4 categories:   1. Patient outcomes: pain, disability, work absence and sickness certification, 2. Safety e.g. serious adverse events, missed red-flag diagnoses/pathologies, 3. Socio-economic and health care costs: consultations, prescriptions, tests, referrals, and impact on GP workload/services. 4. Barriers and facilitators of MSK triage and patient direct access/self-referral services. |  |
| **Setting** | Context was first contact, non-traumatic, non-emergency care, including walk-in centres in primary care and / community care settings Musculoskeletal triage / direct access services offered by non-medical (i.e. non-GP) allied health professionals. | MSK triage / direct access services within secondary and tertiary care were excluded.  Services with more than 40% privately funded/insured patients were excluded  Studies set in A & E or walk-in centres in hospitals were excluded. |
