# Supplementary material for: A systematic review and evidence synthesis of non-medical triage, self-referral and direct access services for patients with musculoskeletal pain
Source: PLoS One. 2020 Jul 6;15(7):e0235364. doi: 10.1371/journal.pone.0235364 (PMC7337346; doi:10.1371/journal.pone.0235364)
Supplement: S3 Table — (DOCX) [file pone.0235364.s004.docx]

S3 Table: Modified GRADE Criteria

| Criteria | - Hierarchy of evidence (https://www.cebm.net/index.aspx?o=5653) in relation to the primary sources of data (e.g. poor quality cohort and cross-sectional observational studies without sensitivity analyses was rated as weak evidence, while good quality cohort or cross sectional analyses of large data sets and well conducted RCTs, were graded as higher level of evidence. - Quality of the evidence (level of methodological quality as assessed by the MMAT checklist) - Data relating to difference in means of outcomes between MSK triage and direct access/self-referral services compared to usual GP led care; and for binary outcomes, differences in rates/amounts of subsequent care episodes - Level of precision (confidence interval and level of significance; p<0.05) - The consistency of results across the body of evidence for each outcome and across the MSK triage and direct access/self-referral service models. |
| --- | --- |
| Grade ratings | 1. “Very weak evidence” - in the absence of empirical data (qualitative or quantitative) on the effect of MSK triage and direct access/self-referral services on patient related outcomes 2. “Limited evidence” - in the presence of evidence from cohort and cross-sectional observational studies, lacking comparisons with usual GP led care, AND when there were small, inconsistent, or non-significant differences in patient related outcomes, OR without sensitivity analyses 3. “Moderate evidence” - in the presence of evidence from good quality cohort and cross-sectional analyses of large data sets, comparisons with usual GP led care, and /or with small to moderate but consistent effects on patient related outcomes as a result of MSK triage or self-referral services. 4. “Strong evidence” - in the presence of evidence from good quality trials, cohort and cross-sectional analyses of large data sets, comparisons with usual GP led care, and /or with moderate to strong consistent effects on patient related outcomes as a result of MSK triage or self-referral services. |
