# Supplementary material for: A systematic review and evidence synthesis of non-medical triage, self-referral and direct access services for patients with musculoskeletal pain
Source: PLoS One. 2020 Jul 6;15(7):e0235364. doi: 10.1371/journal.pone.0235364 (PMC7337346; doi:10.1371/journal.pone.0235364)
Supplement: S4 Table — (DOCX) [file pone.0235364.s005.docx]

*S4 Table: Methodological Quality Appraisal MMAT tool*

| First Author / Publication Year | Quantitative randomised controlled trials | | | | | Quantitative non-randomised study | | | | | Quantitative descriptive | | | | | Qualitative | | | | | Mixed methods study | | | | | |
| --- | --- | --- | --- | --- | --- | --- | --- | --- | --- | --- | --- | --- | --- | --- | --- | --- | --- | --- | --- | --- | --- | --- | --- | --- | --- | --- |
|  | Is randomization appropriately performed? | Are groups comparable @ baseline? | Are there complete outcome data? | Are outcome assessors blinded to the intervention? | Participants adhere to assigned intervention? | Participants representative of the target population? | Measurements appropriate for the outcome & intervention/ exposure? | Are there complete outcome data? | Are the confounders accounted for in the design and analysis? | Is the intervention administered (or exposure occurred) as intended? | Is sampling strategy relevant to the research question? | Is sample representative of the target population? | Are the measurements appropriate? | Is the risk of nonresponse bias low? | Is statistical analysis appropriate to the research question? | Is qualitative approach appropriate to the research question? | Are the qualitative data collection methods adequate to address the research question? | Are the findings adequately derived from the data? | Is the interpretation of results sufficiently substantiated by data? | Is there coherence between qualitative data sources, collection, analysis and interpretation? | Is there adequate rationale for using a mixed methods design for the research question? | Are the different components of the study effectively integrated to answer the research question? | Are the outputs of the integration of qualitative and quantitative components adequately interpreted? | Are divergences and inconsistencies between quantitative and qualitative results adequately addressed? | Do the different components adhere to the quality criteria of each tradition of the methods involve? |  |
| Greenfield 1975 | **!** | **!** | **+** | **-** | **!** |  |  |  |  |  |  |  |  |  |  |  |  |  |  |  |  |  |  |  |  |  |
| Overman 1988 | **+** | **+** | **+** | **!** | **+** |  |  |  |  |  |  |  |  |  |  |  |  |  |  |  |  |  |  |  |  |  |
| Bishop 2017 | **+** | **+** | **-** | **+** | **+** |  |  |  |  |  |  |  |  |  |  |  |  |  |  |  |  |  |  |  |  |  |
| Bornhoft 2015 |  |  |  |  |  | **+** | **+** | **+** | **-** | **+** |  |  |  |  |  |  |  |  |  |  |  |  |  |  |  |  |
| Ludvigsson 2012 |  |  |  |  |  | **+** | **+** | **+** | **+** | **!** |  |  |  |  |  |  |  |  |  |  |  |  |  |  |  |  |
| Mintken 2015 |  |  |  |  |  | **+** | **+** | **!** | **-** | **!** |  |  |  |  |  |  |  |  |  |  |  |  |  |  |  |  |
| Moore 2005 |  |  |  |  |  | **!** | **+** | **!** | **-** | **!** |  |  |  |  |  |  |  |  |  |  |  |  |  |  |  |  |
| Ojha 2015 |  |  |  |  |  | **-** | **+** | **-** | **!** | **+** |  |  |  |  |  |  |  |  |  |  |  |  |  |  |  |  |
| Denninger 2018 |  |  |  |  |  | **+** | **+** | **-** | **!** | **+** |  |  |  |  |  |  |  |  |  |  |  |  |  |  |  |  |
| Goodwin 2016 |  |  |  |  |  | **!** | **+** | **-** | **-** | **+** |  |  |  |  |  |  |  |  |  |  |  |  |  |  |  |  |
| Phillips 2012 |  |  |  |  |  | **-** | **+** | **-** | **-** | **+** |  |  |  |  |  |  |  |  |  |  |  |  |  |  |  |  |
| Holdsworth 2007 |  |  |  |  |  | **+** | **+** | **-** | **-** | **+** |  |  |  |  |  |  |  |  |  |  |  |  |  |  |  |  |
| Swinkels 2014 |  |  |  |  |  | **+** | **+** | **!** | **+** | **!** |  |  |  |  |  |  |  |  |  |  |  |  |  |  |  |  |
| Pendergast 2012 |  |  |  |  |  | **+** | **+** | **+** | **+** | **+** |  |  |  |  |  |  |  |  |  |  |  |  |  |  |  |  |
| McGill 2013 |  |  |  |  |  | **-** | **!** | **!** | **-** | **!** |  |  |  |  |  |  |  |  |  |  |  |  |  |  |  |  |
| Mitchell 1997 |  |  |  |  |  | **!** | **!** | **!** | **-** | **!** |  |  |  |  |  |  |  |  |  |  |  |  |  |  |  |  |
| Mallet 2014 |  |  |  |  |  |  |  |  |  |  | **+** | **+** | **!** | **-** | **!** |  |  |  |  |  |  |  |  |  |  |  |
| Boissonnault2016 |  |  |  |  |  |  |  |  |  |  | **+** | **+** | **+** | **+** | **+** |  |  |  |  |  |  |  |  |  |  |  |
| Badke 2014 |  |  |  |  |  |  |  |  |  |  | **+** | **+** | **+** | **+** | **+** |  |  |  |  |  |  |  |  |  |  |  |
| Scheele 2014 |  |  |  |  |  |  |  |  |  |  | **+** | **+** | **+** | **+** | **+** |  |  |  |  |  |  |  |  |  |  |  |
| Ferguson 1999 |  |  |  |  |  |  |  |  |  |  | **+** | **+** | **+** | **!** | **!** |  |  |  |  |  |  |  |  |  |  |  |
| Mant 2017 |  |  |  |  |  |  |  |  |  |  | **+** | **+** | **+** | **-** | **+** |  |  |  |  |  |  |  |  |  |  |  |
| Harland 2016 |  |  |  |  |  |  |  |  |  |  | **-** | **-** | **+** | **-** | **+** |  |  |  |  |  |  |  |  |  |  |  |
| Pearson 2016 |  |  |  |  |  |  |  |  |  |  |  |  |  |  |  | **+** | **+** | **+** | **+** | **+** |  |  |  |  |  |  |
| Boissonnault 2010 |  |  |  |  |  |  |  |  |  |  |  |  |  |  |  |  |  |  |  |  | **+** | **+** | **+** | **+** | **+** |  |
| Chetty 2012 |  |  |  |  |  |  |  |  |  |  |  |  |  |  |  |  |  |  |  |  | **+** | **-** | **+** | **+** | **!** |  |
| McCallum 2012 |  |  |  |  |  |  |  |  |  |  |  |  |  |  |  |  |  |  |  |  | **+** | **!** | **!** | **-** | **+** |  |
| + : Yes, - : No, ! : Can’t tell | | | | | | | | | | | | | | | | | | | | | | | | | | |

| ***S 5 Table: Classification of Direct access and first contact service models*** | | | | | | | | | | |
| --- | --- | --- | --- | --- | --- | --- | --- | --- | --- | --- |
| ***First Author /Year of publication*** | ***Method of delivery of Triage or Direct access*** | ***Intervention package*** | ***Fidelity of intervention: Planned vs. Actual delivery of the programme*** | ***Expertise, specific training received "for providing service*** | ***Additional notes on relevant features*** | ***Clinical/patient orientated outcome*** | | ***Economic Outcomes*** | ***Barriers*** | ***Facilitators*** |
|  |  |  |  |  |  | ***Pain/Disability*** | ***QOL/adverse effects*** | ***Healthcare utilisation & Work*** |  |  |
| **Model: Open direct access to first contact practitioner (physiotherapists)** | | | | | | | | | | |
| Badke et al 2014 | face to face | DA: non-specified usual physiotherapy care | Pilot initially established and widely implemented. | Specialised training for PTs prior to practicing within the DA model of care | No of sessions (SD)=3.9 (3.0)  Duration of Rx weeks (SD): 10.2 (8.1)  Duration of sessions: NR | % decrease in pain:64.6  Mean functional improvement (SD) : 15.2(11.7) | n/a | Mean total cost of care per patient (SD): $2423.5 (2555.3). | n/a | Reduced costs with no apparent compromise in patient orientated outcomes increased support for model |
|  |  | PR: usual care/Physician referral | 24 care episodes removed from analyses for inpatient stays, special referral request and having both Physician Referral and Direct Access. | n/a | No of sessions (SD)=5.4 (3.2)  Duration of Rx weeks (SD): 8.4(8.6)  Duration of sessions: NR | % decrease in pain:66.6  Mean functional improvement (SD):14.6 (10.6) | n/a | Mean total cost of care per patient (SD): $3878.7 (2923.8) |  |  |
| Boissonault 2010/2016 | face to face | DA: non-specified usual physiotherapy care. | Institutional approval and adjustments to organisation of services. | Specialised Training for PTs. | Standardised decision making protocol was considered for use in PT DA consultations. | n/a | n/a | Referrals for further investigations (e.g. x-ray), pain medications, and physician consultations were initiated for <30 %of DA patients | Legality, institutional policies and restrictions.  Staffing models and operational issues  Patient health plans necessitating PR  Lack of awareness (patients) or knowledge regarding potentials of DA.  Smaller/ rural providers were less likely to offer DA. | DA promoted to staff who field patient calls.  However, only 33% to 50% of designated direct access patient scheduled slots.  Thus further internal and external marketing strategies such as newsletters, community wide promotion had to be initiated. |
|  |  | PR: non-specified usual GP care and physiotherapy referral if needed. | direct access patient care slots were identified on therapists’ schedules on days when physicians were physically on-site | n/a | n/a | n/a | n/a | n/a |  |  |
| Mintken 2015 | face to face | DA: non-specified usual physiotherapy care. | Patients with spine & rib pain were initially exempted for part of the time under review | Advanced degrees and board certifications | No / duration of sessions & Duration of Rx weeks (SD) : NR | n/a | No serious adverse events | No of patients seeing PTs after referral from physicians reduced to 46% over the 10yr period. | n/a | Fast-tracked access to physiotherapy |
| Moore 2005 | face to face | DA: non-specified usual physiotherapy care. | n/a | PTs with an average (SD) 7.4 (9.7) years’ experience and certifications | n/a | n/a | No serious adverse events | n/a | n/a | DA PTs credentialed to order laboratory and diagnostic imaging  studies, electromyography and/or nerve conduction  studies, and to prescribe medications |
| Ojha 2015 | face to face | DA: Physiotherapy care informed by the Orebro score/protocol. | Intervention informed by the Orebro score | Doctor of Physiotherapy and independent practitioner | No of sessions: 3.7 (1.06)  Duration of sessions: NR  Duration of Rx weeks (SD) : 2 (0.45) | mean increase in functional score 3.6 (range: 5.7 to 9.3) | n/a | Total cost care per patient $435 (140) per visit.  Mean absent work days: 0  Mean restricted work days: 0 | n/a | $75 gift card to incentivize  participation and increase retention |
|  |  | PR: non-specified usual care | n/a | n/a | n/a | n/a | n/a | Mean absent work days 57.9 days (127) Mean restricted work days 8.5 days (24) |  |  |
| Denninger 2018 | face to face | DA: Active physical therapy incl. spinal manipulation, therapeutic exercise and patient education.  NB: on site physician available for advise | Screening protocol to check for red flags and inform DA Guideline informed Programme. Data included those 'on protocol' regarding number of sessions and providing follow-up data. 156 of 603 not included in analysis. Uptake of 38.3% (171 of 447 patients) | 1-day training for Therapists: medical  screening, treatment-based classification,  clinical guidelines for neck and  back pain, and clinical progression. | No of sessions (SD)= 6.6 (5.7, 7.5)  Duration of Rx weeks: 8.4 average  Duration of sessions: NR  Care was provided by same physical therapists but route of access was the difference i.e. via traditional medical referral | Mean decrease in pain (SD): 2.0 (1.6, 2.4)  Mean disability at discharge (SD): 5.6 (4.5, 6.7) | QOL measured at baseline but outcome data NR.  No related adverse events | Total cost care per patient (SD): 1542 (108, 2976).  Average  No quantification of burden of cost of extra advice from physicians. | n/a | n/a |
|  |  | PR: Same physical therapy programme but via physician referral not direct access | n/a | Same as for intervention | Mean no of sessions=7.6 (6.8, 8.3)  Duration of Rx weeks: 10.3 average  Duration of sessions : NR | Mean decrease in pain (SD): 2.0 (1.7, 2.3)  Mean disability at discharge (SD): 6.1 (5.2, 6.9) | No related adverse events | Total cost care per patient (SD): 3085 (1939, 4224) |  |  |
| Swinkels 2014 | face to face | DA: non-specified usual physiotherapy care. includes 10 minute screening protocol to determine eligibility for physio and contraindications | Both referred and direct access received usual physio care. Year on year increase in % episodes for which patients used direct access (2006 28.9% up to 52.1% in 2009) | National protocol encourages exchange of information between PTs and GPs but does not impact autonomy of practice | No of sessions (SD)=8.1 (6.6)  Duration of session & duration of Rx: NR | NR | NR | Average no of subsequent PT visits for DA lower compared to PR | n/a | National adoption  Awareness & education |
|  |  | PR: non-specified usual GP care and physiotherapy referral if needed. | n/a | n/a | No of sessions (SD)=10.5 (8.9)  Duration of session & duration of Rx: NR | n/a | n/a | Increase in PT services via DA but no decrease in volume of care for GP |  |  |
| Pendergast et al 2012 | Face to face in PT’s office or hospital based outpatient PT | DA: non-specified usual physiotherapy care. | NR | NR | Mean no of sessions= 5.90 (5.55)  Duration of sessions & Duration of Rx weeks (SD) : NR | Outcomes of care NR | n/a | There was no difference found in the two groups in the 60 days afterward | n/a | n/a |
|  |  | PR: non-specified usual GP care and physiotherapy referral if needed. | NR | NR | Mean no of sessions= 7.00 (6.09)  Duration of sessions & Duration of Rx weeks (SD) : NR | n/a | n/a- | Total health care cost were higher in PR group, with a difference of about $44 more for men, $18 for women before PT episodes and a difference of ~$70 and $93 more for females & males during PT episodes. |  |  |
| McGill et al 2013 | face to face | DA: Non specified but appear to assess, and manage as per usual physiotherapy care | NR | NR | No/duration of sessions & Duration of Rx weeks (SD) : NR | NR | NR | Medication use: 24.07%  Radiology use: 11.11%  > 50% higher return to duty rates compared to physicians care. | Most therapists were not credentialed to order medication and imaging investigations. | n/a |
|  |  | PR: non-specified usual GP care | NR | NR | No/duration of sessions & Duration of Rx weeks (SD) : NR | NR | NR | Medication use: 90.53%  Radiology use: 82.11%  No significant difference between groups for number of visits. |  |  |
| Holdsworth et al 2007 | face to face | DA: assess, and manage as per usual physiotherapy care | Standard physiotherapy care was not protocolised. Not reported whether physios could refer to secondary care. | n/a | **Duration of Rx weeks (SD):** NR  Physiotherapists via but GP-suggested referral or GP-referral | Mean VAS @baseline :56.8(23.2) and @follow-up: 18.5 (21.7).  No significant difference in clinical outcomes between DA & PR groups | Patient treatment goals achieved @12 months : 67% | Total average cost per episode: £88.9  DA reported nearly half the level of absence at work compared to GP or GP-suggested referrals (2.5 days versus 6 and 6 days).  NB: An examination of the actual number of days absent differed between patient groups. | Patient/ public general lack of awareness and knowledge of Physiotherapy.  Lack of public campaigns and awareness of DA.  Operational constraints specifically service delivery times.  Lack of PTs involved in monitoring and prescribing NSAIDs, issuing sickness certificates and requesting X-rays | Easier and faster access for patients  Experience, confidence and ability levels of PTs.  Specialised training for PTs  GPs support of DA |
|  |  | PR: non-specified usual GP care and physiotherapy referral if needed. | GP suggested referrals were described separately. | n/a | No of sessions for GP-suggested/referral =4 average | Mean VAS @baseline :55.0(23.6) and @follow-up: 18.5 (22.0) | Patient treatment goals achieved @12 months: 65% | Total average cost per episode: £66.31  DA were less likely to be prescribed analgesics and NSAIDs 9.7%, vs. 15.6%; referred for X-ray (7.3% vs 13.6%) or to secondary care than patients referred by their GP (3.1% vs 1.4%). |  |  |
| Goodwin 2016 /Moffatt 2017.  Qualitative data on barriers and facilitators from Moffatt et al. | face to face | Up to 2 consultations, 20 mins in length, with physiotherapist to match normal GP care. Within the physiotherapy assessment patients were screened for non-MSK pathology and, where appropriate, offered advice and any relevant interventions, primarily based within a self-management paradigm. If patients were felt to require on-going physiotherapy input they were referred to the main primary care physiotherapy provider at their second appointment. | NR | PT's were trained on clinical knowledge and spotting red flags | No/duration of sessions & Duration of Rx weeks (SD) : NR | NR | No related adverse events  EuroQol EQ-5d @ 6 months- mean diff (SD):  Inner city practice 0.13(0.27).  University Practice 0.10(0.14). | Mean cost per episode of care  Practice 1: Physio led =£84.26, GP =647.16. Practice 2: Physio led £56,51, GP led £366.44 | GP's believe FCP's will not reduce their workload significantly.  Cultural attitude that patients feel they need GP approval to see the PT.  FCP was feared to lead to de-skilling of GP's.  worries about the NHS wanting to use 'Cheaper' more junior staff was expressed. | Dedicated DA time slots for PTs  Behaviour- patients appeared to be more satisfied with their assessment from a FCP.  Some patients appeared quick to pick up on GP's lack of MSK skills and are likely to ask to be referred to a specialist.  physiotherapists were fully integrated within the primary care team and shared  access to the electronic patient records which were  visible to all providers. |
| Greenfield | Face to face | DA: Nurse led assessment and management plans for back pain | Specific to back pain only  Use of a standardised protocol for assessment and developing management plan | All management plans reviewed by physician supervisor | No/duration of sessions & Duration of Rx weeks (SD) : NR | NR | No serious adverse events @4 months follow up.  Patient satisfaction with improvement: 73% | Radiology use: n= 21 | n/a | n/a |
|  |  | PR: non-specified usual GP care | NR | NR | No/duration of sessions & Duration of Rx weeks (SD) : NR | NR | Patient satisfaction with improvement: 68.5% | Radiology use: n= 36 |  |  |
| Overman et al 1988 | face to face | DA: assessment, diagnosis, advice, onward referral | Use of checklists and algorithm for triage by intern PTs | Specialised training on spinal pain management for DA PTs | No of session: 1. Duration of sessions =33min  Duration of Rx weeks (SD): n/a | Sickness impact profile score (pain & disability): better improvement for those with severe dysfunction at baseline and Physio led. | No adverse outcomes  Less reoccurrence of symptom at follow up compared to PR (24 vs. 44%) | Significantly less medications (analgesics 10 vs. 42% and muscle relaxants) compared to PR | Health Policy/ Legislation | Training  Specific MSK condition- back pain? |
|  |  | PR: assessment, diagnosis, advice, onward referral | Use of checklist |  |  |  |  | Significantly more visits and average time in PT care compared to DA. |  |  |
| Mitchell et al 1997 | face to face | DA: Non specified usual physiotherapy care | Episodes of care defined by physical medicine procedures included non-PTs such as chiropractors | NR | Mean no of sessions= 7.6 (9.1)  Duration of sessions & Duration of Rx weeks (SD) : NR | NR | NR | Total claims: $1004±2030  Number of Therapists visits less by 65% for direct access compared to usual care.  Total claims for all services and drugs less by 135% for direct access compared to usual care. | n/a | n/a |
|  |  | PR: GP usual care with referral or non-referral for physiotherapy. | NR | NR | Mean no of sessions= 12.2 (12.8)  Duration of sessions & Duration of Rx weeks (SD) : NR | NR | NR | Total claims: $2236±2827 |  |  |
| ***First Author /Year of publication*** | ***Method of delivery*** | ***Intervention package*** | ***Fidelity of intervention: Planned vs. Actual delivery of the programme*** | ***Expertise, specific training received "for providing service*** | ***Additional notes on relevant features*** | ***Clinical/patient oriented outcome*** | | ***Economic Outcomes*** | ***Barriers*** | ***Facilitators*** |
|  |  |  |  |  |  | ***Pain/Disability*** | ***QOL/adverse effects*** | ***Healthcare utilisation & Work*** |  |  |
| ***Model: Combination of triage process with open access to first contact (non-medical) practitioner*** | | | | | | | | | | |
| Chetty et al 2012 | Telephone triage (nurse led) ± face to face ? | DA: by triage to different professionals e.g., GPs, PTs, Psychologists | NR | NR | One-time service for triage only.  Localised to one occupational setting. | n/a | n/a | n/a | Inadequate assessment and triage skills/ lack of knowledge of MSK by triage staff  Poor staffing, attitude and organisation of triage service  Too narrow focus on impact of MSK pain on work rather than whole person (occupational setting). | organisation support for evidenced-based practice  Multidisciplinary practice and effective communication strategies.  Triage staff training  Clinical and cost effectiveness of Triage |
| Mallet 2014 | Telephone triage + face to face treatment | DA: assessment, diagnosis, treatment (including further referrals). Usual Physio care, layered with initial physio telephone assessment /appropriateness check by a senior physio. | Low numbers of true Self-Referral uptake by patients.  Initiated by telephone contact from the patient to clerical staff followed by a telephone triage appointment and then face-to-face follow-up appointments. | SR pathway education for GPs, practice managers and primary care staff was undertaken. | No/duration of sessions & Duration of Rx weeks (SD) : NR | Mean pain @discharge: 74.67, ±18.0 | Waiting time satisfaction score: 74.7% | Total cost care per patient per visit (SD): £434 803  Mean waiting time of 3.55 days compared with 30.99 days in the usual care group for 1^st^ Physio assessment. | Authors attribute to lack of resources for advertising and an ongoing culture of GP reliance for MSK management. | Patient information leaflets and DA advertisements were placed in all participating GP surgeries. |
|  |  | PR: Usual care- Referred by GPs to physiotherapist as needed. | NR | NR | NR | Mean pain @discharge VAS score : 74.67, ±18.0. | Waiting time satisfaction score: 53.2% | Total cost care per patient per visit (SD): £645 773. |  |  |
| Bornhoft 2015 | Telephone  /face to face | DA: Initial Physio assessment, diagnosis and treatment | Focus on the triaged system. Fidelity was described by the book but not evaluated.  Follow up difficulties could limit fidelity of the analysis | NR | No/duration of sessions & Duration of Rx weeks (SD): NR | Mean pain @discharge VAS score : 77.49, ±17.84 | NR | Further GP visits (%): 221 (38.0)  Further referrals to specialists/examinations (%): 101 (17.4)  Sick-leaves (%): 82 (14.1)  Further Prescriptions (%): 129 (22.2) | Referrals to specialists e.g. orthopaedics, rheumatology and neurology, or to pain clinics were limited to medical specialists.  Age and depression often affected outcome | n/a |
|  |  | PR: Initial contact with nurse (tel, F2F) who booked patients to see GP. Initial assessment, diagnosis and/or treatment by GP prior to referral if needed | n/a | NR | Care provider: Physician/GPs- face to face | Mean pain @discharge VAS score : 77.49, ±17.84 | NR | Further GP visits (%): 777 (48.8)  Further referrals to specialists/examinations (%): 636 (39.9)  Sick-leaves (%): 369 (23.2)  Further Prescriptions (%): 1156 (72.6) |  |  |
| Phillips 2012 | Telephone +/-  face to face | DA: Physiotherapist telephone advice and triage to provide rapid, easily accessible advice and signposting to relevant services | Uptake of service was only 54% of that anticipated beforehand. | Specialised training was provided to involved PTs | No/duration of sessions & Duration of Rx weeks (SD): NR | Mean pain @discharge VAS score: 6.91 (9.4) significantly lower that @ baseline: 10.54 (9.4).  Mean Functional outcome measured by General health Questionnaire : 10.11 (5.7) significantly better @ follow up compared to baseline 12.95 (6.1) | Mean QoLmeasured by EQ5D score: 0.82(0.2) significantly better than baseline 0.66 (0.2) | Mean sickness absence: 1.45 (9.7) significantly lower compared to baseline 4.6 (12.6)  Mean work performance index: 87.8 (13.2) significantly better than baseline 75.9 (19.6) | Telephone advice and workplace assessment pathway less successful: operational issues? | Continuous high level promotion and advertisement irrespective of lower than anticipated service uptake |
| Ferguson et al 1999 | Telephone +/-  face to face | DA: PTs accessed brief report of patient symptoms and then contacts by telephone for further assessment and consultation as appropriate. | High uptake  Limited to patients with <2 months onset of symptoms | liaison with GPs where required | No of sessions: mode= 5  duration of sessions & Duration of Rx weeks (SD): NR | NR | NR | NR | Operational issues: inundated requests  Pt expectation of immediate access to a PT. | Public awareness and advertisements  PT access to patient records  Dedicated time slots for DA |
| Ludvigsson 2012 | Telephone +/-  face to face | DA: mainly primary assessment plus management | Fidelity of triage not reported. uptake of Physiotherapy appointment were as per triaged by nurses | not specified | No/duration of sessions & Duration of Rx weeks (SD**) :** NR | EQ VAS @3months Mean (SD): 67(18) | EQ 5D @3months Mean (SD): 0.65 (0.22) | Further GP referrals: 12%  Further referral to physiotherapy or GP: 29% | n/a | n/a |
|  |  | PR: assessment, diagnosis plus medical prescriptions plus PT referral where indicated | Usual patient flow management | n/a | n/a | EQ VAS @3months Mean (SD):  56 (19) | EQ 5D @3months Mean (SD):  0.51 (0.3) | Further medical referrals: 48%  Medical prescriptions : 60%), X-rays: 29%, laboratory tests: 24% and medical (sickness) certificates 17%. | n/a | n/a |
| ***First Author /Year of publication*** | ***Method of delivery of Triage or Direct access*** | ***Intervention package*** | ***Fidelity of intervention: Planned vs. Actual delivery of the programme*** | ***Expertise, specific training received "for providing service*** | ***Additional notes on relevant features*** | ***Clinical/patient oriented outcome*** | | ***Economic Outcomes*** | Barriers | Facilitators |
|  |  |  |  |  |  | ***Pain/Disability*** | ***QOL/adverse effects*** | ***Healthcare utilisation & Work*** |  |  |
| ***Service Based Pathways*** | | | | | | | | | | |
| Bishop et al 2017 | NB: non patient level intervention:  Cluster trial where many people in the analysis did not receive any form of physiotherapy. DA was face to face where appropriate | DA: Telephone assessment, education, and advise ± further Physiotherapy intervention. | Of the 152 patients in the intervention arm, only 44 were "true" self-referral. others were recommended.  Direct access pathway was used by 90% of patients in intervention practices needing physiotherapy. | Training | **No/duration of sessions & Duration of Rx weeks (SD) : NR** | Disability (SF 36 physical component score) | EuroQol EQ-5d @ 12 months: 0.606 (0.258)  No adverse events or safety issues identified. | Total cost care per patient (SD): 940.02 (2157.24).  An overall increase in the number of referrals to physiotherapy in one intervention arm practices compared with service-level data in the previous year. | - | Similar clinical and  cost outcomes in both groups |
|  |  | PR: Non-availability of self-referral pathway. Usual care- Referred by GPs to physiotherapist as needed. | NR | No training | **No/duration of sessions & Duration of Rx weeks (SD) : NR** | - | EuroQol EQ-5d @ 12 months: 0.615 (0.254)  No adverse events or safety issues identified. | Total cost care per patient (SD): £951.25 (2050.88)  Waiting times for physiotherapy did not increase (28 days before, 26 days after introduction of direct access).  slightly more GP visits, more investigations (including scans, X-rays and MRIs) and visits to consultants (rheumatologists and orthopaedic surgeons) and inpatient days for surgeries. | n/a | n/a |

PTs: Physiotherapists, DA: Direct Access, PR: Physician referred, NR: not reported, MSK: musculoskeletal
