# Supplementary material for: A systematic review and evidence synthesis of non-medical triage, self-referral and direct access services for patients with musculoskeletal pain
Source: PLoS One. 2020 Jul 6;15(7):e0235364. doi: 10.1371/journal.pone.0235364 (PMC7337346; doi:10.1371/journal.pone.0235364)
Supplement: Attachment — Submitted filename: Response to Reviewers MSK Triage Evidence Synthesis.docx [file pone.0235364.s007.docx]

**28^th^ May 2020**

Dear Academic Editor and Editor-In-Chief,

Many thanks for your consideration of our manuscript.

We would like to thank the reviewers for their helpful feedback on our manuscript. Responses to each of the reviewers’ comments are addressed below. Within the revised manuscript, changes to original text have been included as tracked changes.

We hope you will find the revised manuscript acceptable for publication in PLOS ONE.

Kind regards,

Dr. Opeyemi Babatunde

On behalf of all authors.

***Response to Reviewer’s comments***

***Reviewer #1***:

This article researches the evidence regarding characteristics, outcomes, barriers and facilitators of musculoskeletal (MSK) triage and direct access services using meta-analysis technique. This view point is nice and collected data was huge volume. The results demonstrated that methods and outcomes of care did not much difference between GP-led care and direct access. However, studies were so heterogeneous that statistical analysis was not fulfilled in the article. I think this article is worth publishing in Plos One because much information is in the article.
One thing authors should put full spelling of MSK before first appearance in ABSTRACT.

***Authors’ response:*** We thank reviewer #1 for commendation of this work and manuscript. Full meaning of MSK has now been spelt out in the abstract.

***Reviewer #2:***

This paper reports the existing evidence regarding characteristics, outcomes, barriers and facilitators of MSK triage and direct access services. There is also a substantial health economics outcome section, which is reported separately to the clinical outcomes. The review was conducted using several established databases (although only 3 are reported in the abstract). The attempt was for a more quantitative piece but the quality of the research has produced a narrative synthesis review focusing on three areas. The findings include suggestions that Direct Access patients have similar characteristics as GP led services, there is a mixed type of model used, the barriers and facilitators are identifiable and again varied. Finally little difference was shown in outcomes (clinical) but limitations in design restrict such conclusions. This paper has much to commend it; namely

• The subject is topical and is a growing area and will therefore of interest to the MSK readership.

• There is little work on this area to date.

• The work is very well executed and well written, with high levels of rigour and balance.

• All the correct instruments for systematic review have been used; it is registered on Prospero and reported according to PRISMA guidelines.

• Resolution of conflicts for each item was done appropriately.

• The work on healthcare economics is more persuasive and perhaps one of the stronger messages (acknowledged by the authorship team).

• The Discussion is reasonable and clear.

• Limitations are fairly reported.

• The Conclusion is sensible.

Despite these qualities there also some (perhaps more minor) areas of disquiet.

• The review is only a narrative review which does limit its use. (It is accepted this is not the research teams’ issue)

• There was inclusion of experimental studies only (presumably with a view to an attempt at a more quantitative piece in the first instance – which was not possible). This may have limited some rich information which would have been acceptable in a narrative review.

• Barriers and facilitators reflect largely administration issues• One of my substantial concerns is the overall gravity and impact of the work. The first two aims are a little lacking in substance - describing characteristics of the population (just age and gender) and some chronicity does not really help practice a great deal. Similarly, describing the models available is also a little lacking in ambition. The work on outcomes has more of a research question feel and would appear to offer greater impact. However, with only 9 studies included and an inability to fully synthesise data, any useful conclusion becomes difficult (again to be entirely fair to the authors they acknowledge this).

• Some of the tables are unwieldy (I understand they are necessary to some extent for Reviews). Table 2 will be unpublishable because of the amount of whitespace and limited information provided.

• Table 3 has a great deal more information than just the type or model of self-referral. Again unlikely to be publishable in the given form.

Summary: A well-executed piece of work and well written up. It is a valuable resource of information on this subject and will help others in the area but, the impact of the work could be questioned. Systematic reviews are useful if they increase our understanding or change practice. There is some modest increase in our understanding but impact on practice is unlikely. As an information compendium and assimilation of available literature it is high quality and therefore does has value.

***Authors’ response:*** The team is similarly grateful for reviewer #2’s commendation of our manuscript as well as the helpful comments. Our comprehensive search of 8 databases (of which 3 was named in the abstract) was stated as such for succinctness. Based on our knowledge of the field and the specific aims of the systematic review, we envisaged a priori a mixed methods/narrative synthesis of data. This was stated in the protocol and PROSPERO registration document. Therefore, drawing on all available evidence from experimental, quasi-experimental and qualitative studies, we conducted a narrative evidence synthesis in a systematic and structured manner as outlined in the methods section (page 3-4) of the manuscript. Inherent heterogeneity (expected) in the data presented across included studies precluded planned meta-analysis and necessitated a narrative synthesis approach (*Popay, Jennie, et al. "Guidance on the conduct of narrative synthesis in systematic reviews." A product from the ESRC methods programme Version 1 (2006): b92*).

As per eligibility criteria established a priori (please see page 2 of the manuscript), this systematic review and evidence synthesis includes both experimental (quantitative) and non-experimental (e.g. qualitative) studies.

The team feel the stated objectives of the review are relevant. Given that available literature (page 1, line 59-63; references 7-10) on this topic shows that direct access services were more likely to be used by select groups of patients. This review aimed to ascertain if this is true across all currently available models of care and also explore ways by which MSK triage and direct access services (if beneficial for patient outcomes such as pain and functional disability) could be improved and extended to all, thereby decreasing health inequality. Further reflections on the first two objectives of the review and related findings are highlighted in page 18 lines 420-423 of the revised manuscript.

We share reviewer #2’s observation regarding barriers and facilitators reflecting largely administrative issues and we feel this is an important finding of this review. Much of the research aiming to inform clinical practice have often failed to address issues related to organisation and access to care. Potentially, large gains in patient satisfaction and patient-oriented clinical outcomes may be gained as a result of simple cost effective solutions relating to the admiration and organisation of care. Please refer to the last paragraph of the discussion section (page 24) for this reflection in the revised manuscript.

Table 2 & 3 has now been offered as supplementary files.

***Reviewer #3***:

Your study is a successful one and contribute the literature an important subject. I have a few offer, thank you.

1. You start with MSK in the abstract, please do not use abbrevation wşthout defining it before.
2.You need to define aim of the study better.,
3. Thi fügure 1 do not seem clear. Also you have an extra space between the numbers such as 13 176; 9 010. Please delete the unnecessary spaces.
4.Your reference number is few. This is a review study and I think you should use more studies.

***Authors’ response: Thank you.***

1. We apologise for this omission and the term MSK has now been spelt out at first mention in the abstract.
2. We have tried to further clarify the objectives of the review in the revised introduction section (page 1-2), also in response to comments by reviewer #2.
3. Figure 1 has now been amended to better reflect the search results as presented within the manuscript (please refer to revised version of Fig 1).
4. We have been careful to use key and pertinent literatures in this field within the manuscript.

***Additional Editor comments/Journal Requirements:***

In the revised manuscript, we have ensured adherence to PLOS ONE's style throughout, and included large tables as supplementary files.

In the methods section (page 2), we have clarified our awareness of more recent articles published since February 2018 when the main searches for our review was conducted. In addition to this, a search update was conducted in May 2020. Having screened 720 citations and abstracts, 15 full texts, we have found only four studies that are potentially eligible. These new studies however do not change the findings of the current review.
